# Inappropriate sales of hypochlorous acid solution in Japan: An online investigation

**DOI:** 10.1017/ice.2020.1248

**Published:** 2020-10-08

**Authors:** Satoru Mitsuboshi, Ryo Yamaguchi, Hiroyuki Uchida, Satoshi Kamoshida, Hideki Hashi

**Affiliations:** 1 Department of Pharmacy, Kaetsu Hospital, Niigata, Japan; 2 Department of Pharmacy, The University of Tokyo Hospital, Tokyo, Japan; 3 Department of Pharmacy, Kanto Rosai Hospital, Kanagawa, Japan; 4 Department of Pharmacy, Mito Brain Heart Center, Ibaraki, Japan; 5 Department of Pharmacy, Tokyo Bay Urayasu Ichikawa Medical Center, Chiba, Japan

*To the Editor—*The use of disinfectants has become more widespread due to the spread of severe acute respiratory coronavirus virus 2 (SARS-CoV-2). In particular, chlorine-based disinfectants such as hypochlorous acid solution (HAS) are inexpensive and widely available. The World Health Organization and the US Centers for Disease Control and Prevention state that HAS should be used only for products and environmental surfaces and is not suitable for external or internal use by humans or for fogging of indoor spaces.^1,2^ This chlorine gas carries a risk of adverse respiratory system events, even at low levels of exposure.^3,4^ However, adverse events associated with disinfectants have been increasing in the United States since January 2020; 62% of these were caused by chlorine disinfectants, with inhalation as the main factor.^5^ Accordingly, adverse events related to the inappropriate use of disinfectants may be increasing worldwide.

In May 2020, the Japanese Ministry of Economy, Trade, and Industry warned the public that HAS should not be used for fogging or for direct application onto the hands.^6^ Because the supply of alcohol-based disinfectants began to run low as SARS-CoV-2 spread in Japan, many facilities (eg, nursery schools, elementary schools, railway companies, restaurants, and public institutions) introduced devices to atomize HAS, inappropriately using it to fog indoor spaces or to directly sanitize the hands. Although HAS products have been sold by many companies, these products may have been sold without information on their appropriate use. Here, we investigated the inappropriate sale of HAS by companies in Japan.

We searched the Amazon website (https://www.amazon.co.jp/) for the term “hypochlorous acid solution” on August 1, 2020, and assessed all companies identified. The Amazon website was used to investigate the provision of inappropriate product information, inappropriate use recommendations, and inappropriate advertising. When a company listed its website, we also investigated the site. The details of the items investigated are shown in Table 1. Companies that did not sell HAS products were excluded. We assessed whether efficacy and safety examinations had been performed based on the recommendations of one of several public institutions, including the International Organization for Standardization, Japanese Industrial Standards, the European Committee for Standardization, the American Society for Testing and Materials, or the Association of Official Analytical Chemists. In total, 97 companies were investigated.

**Table 1.** Inappropriate Sales of Hypochlorous Acid Solution (N = 97)

Variable	No. (%)
**Provision of inappropriate product information**	
Product concentration, no	30 (31)
Product pH, no	54 (56)
Product expiration date, no	54 (56)
Removal of dirt before use of disinfectant, no	91 (93)
Correct amount to use, no	91 (93)
Efficacy examination by public institution, no	97 (100)
Safety examination by public institution, no	97 (100)
**Inappropriate use recommendations**	
Use by spraying, yes	91 (93)
Use by direct application onto hands, yes	39 (40)
Use by fogging of indoor spaces, yes	58 (59)
**Inappropriate advertising**	
Use of the word “disinfection,” yes	32 (33)
For use in healthcare facilities, yes	20 (21)

Our research revealed that many companies provided inappropriate product information, recommended inappropriate use, and engaged in inappropriate advertising practices. To our knowledge, this is the first report to clarify the inappropriate sale of HAS products. Three concerns regarding efficacy, safety, and hype were raised by these results.

First, the efficacy of many HAS products could not be assessed using standard indicators. For example, 30% of products had no label information on concentration, and 50% of products had no label information on pH or expiration date. In addition, most products did not describe the removal of dirt before the use of the disinfectant and the correct amount to use. Previous reports suggest that the presence of dirt, such as organic matter, and the use of an insufficient amount decrease the effectiveness of chlorine-based disinfectants and that their effectiveness may decrease at 2–4 weeks after the container has been opened.^7,8^ Moreover, it was unclear whether any of the products had undergone standard tests performed by public institutions to determine their safety and efficacy.^9^ HAS products may be useful for preventing the transmission of SARS-CoV-2 when they are used at the appropriate concentration and in the appropriate manner. However, these products may not have been effective for such an application.

Second, these products may cause adverse events. More than 90% of the products were recommended to be used by spraying, and ~50% of them were recommended to be used by direct application onto the hands or by fogging of indoor spaces. Chlorine gas has a risk of adverse respiratory system events.^3^ Children, the elderly, and individuals with comorbidities, all of whom have low toxicity tolerance, may be at particularly high risk of adverse events from exposure to chlorine gas resulting from inappropriate use of HAS.^3,4^ Indeed, some adverse events caused by HAS have been reported in Japan, possibly reflecting its inappropriate use.^10^

Third, 33% of products were advertised using the word “disinfection.” The use of the word “disinfection” is allowed only for medications registered under the Pharmaceutical and Medical Device Act in Japan. No HAS products are registered as medications in Japan, and thus such advertisements may be illegal. Moreover, 20% of the products were advertised for use in healthcare facilities. The reference to healthcare facilities may give people false reassurance concerning the effectiveness of the products and may be a strategy to boost sales. HAS may be inappropriately used in healthcare facilities that lack disinfectant specialists, such as nursing homes, clinics, and dental clinics. Further strategies for the proper use of disinfectants in these facilities may be needed.

Our research has some limitations. Only those companies listing products on the Amazon website were studied; thus, these results need to be confirmed by further research. However, many people use the Amazon website because of its prominence. In addition, because we investigated only those products that contained HAS, other products that were inappropriately advertised were not included in our investigation.

In conclusion, to protect patients and the general public from health hazards associated with the inappropriate use of HAS, it may be necessary to develop and implement appropriate regulations and to conduct awareness campaigns for both healthcare providers and the general public.
